# Radiomics Study for Predicting the Expression of PD-L1 and Tumor Mutation Burden in Non-Small Cell Lung Cancer Based on CT Images and Clinicopathological Features

**DOI:** 10.3389/fonc.2021.620246

**Published:** 2021-08-06

**Authors:** Qiang Wen, Zhe Yang, Honghai Dai, Alei Feng, Qiang Li

**Affiliations:** Department of Radiation Oncology, Shandong Provincial Hospital Affiliated to Shandong First Medical University, Jinan, China

**Keywords:** radiomics features, computed tomography, non-small cell lung cancer (NSCLC), programmed death-ligand 1 (PD-L1), tumor mutation burden (TMB)

## Abstract

**Background:**

The present study compared the predictive performance of pretreatment computed tomography (CT)-based radiomics signatures and clinicopathological and CT morphological factors for ligand programmed death-ligand 1 (PD-L1) expression level and tumor mutation burden (TMB) status and further explored predictive models in patients with advanced-stage non-small cell lung cancer (NSCLC).

**Methods:**

A total of 120 patients with advanced-stage NSCLC were enrolled in this retrospective study and randomly assigned to a training dataset or validation dataset. Here, 462 radiomics features were extracted from region-of-interest (ROI) segmentation based on pretreatment CT images. The least absolute shrinkage and selection operator (LASSO) and logistic regression were applied to select radiomics features and develop combined models with clinical and morphological factors for PD-L1 expression and TMB status prediction. Ten-fold cross-validation was used to evaluate the accuracy, and the predictive performance of these models was assessed using receiver operating characteristic (ROC) and area under the curve (AUC) analyses.

**Results:**

The PD-L1-positive expression level correlated with differentiation degree (p = 0.005), tumor shape (p = 0.006), and vascular convergence (p = 0.007). Stage (p = 0.023), differentiation degree (p = 0.017), and vacuole sign (p = 0.016) were associated with TMB status. Radiomics signatures showed good performance for predicting PD-L1 and TMB with AUCs of 0.730 and 0.759, respectively. Predictive models that combined radiomics signatures with clinical and morphological factors dramatically improved the predictive efficacy for PD-L1 (AUC = 0.839) and TMB (p = 0.818). The results were verified in the validation datasets.

**Conclusions:**

Quantitative CT-based radiomics features have potential value in the classification of PD-L1 expression levels and TMB status. The combined model further improved the predictive performance and provided sufficient information for the guiding of immunotherapy in clinical practice, and it deserves further analysis.

## Introduction

Non-small cell lung cancer (NSCLC) accounts for 75%–85% of all lung cancers. Approximately 80% of NSCLC cases are diagnosed at an advanced stage, when curative surgery is not an ideal option (1). Systemic platinum-based doublet chemotherapy is the standard treatment strategy and may provide survival benefits for locally advanced patients, with progression-free survival (PFS) of 3.6–4.8 months and median overall survival (mOS) ranging from 7.9 to 10.3 months (2). These limited and unsatisfactory survival times have necessitated the development of novel treatment modalities, such as immunotherapy, for patients with advanced-stage NSCLC (3).

Immunotherapy is changing the therapeutic strategy for NSCLC to immune checkpoint inhibitors (ICBs). Programmed cell death protein 1 (PD-1) and its ligand programmed death-ligand 1 (PD-L1) play crucial roles in tumor immune escape and the development of the tumor immune microenvironment and closely correlate with tumor generation and invasion (4). Antibodies binding on the PD-1/PD-L1 have been identified in NSCLC patients who were not sensitive to platinum-based chemotherapy (5, 6). Accumulating evidence confirmed that blocking this pathway reversed the immune escape microenvironment and improved the endogenous antitumor immune response (7). The PD-L1 expression level is a commonly used biomarker that indicates whether a patient should receive ICB. Lung cancer patients with PD-L1 positivity are more sensitive to immunotherapy (8, 9). In addition, PD-L1 expression in the early stages of NSCLC could be used as a predictive biomarker for subsequent therapies (10). However, some studies declared an opposite viewpoint. As Diggs et al. (11) and Hirsch et al. (12) reported, PD-L1-negative or low-positive patients also had a good response to antibodies due to high tumor heterogeneity (13). Therefore, PD-L1 expression status alone is not treated as a predictive biomarker of response but rather a useful risk factor for identifying patients who are more likely to benefit from anti-PD-1/PD-L1 monotherapy. Several other candidate predictive biomarkers were associated with clinicopathological factors, the tumor microenvironment, and tumor-infiltrating cells. Tumor mutation burden (TMB), as an important predictive surrogate biomarker of prognosis and response to immunotherapy, has been widely investigated in clinical trials (14–16). Hellmann et al. (17) and Rizvi et al. (18) indicated that higher TMB correlated with favorable outcomes using next-generation sequencing (NGS) assay, which support its potential predictive value. Gandara et al. (19) also suggested that higher TMB in tissues was associated with efficacy of first-line immunotherapy, and TMB in blood predicted the response in NSCLC patients treated with ICB as a second-line treatment. Currently, the gold standards for the detection of PD-L1 expression and TMB are biopsy specimens or surgical resection, which are limited in patients in poor condition due to the invasiveness, time-consuming, tumor heterogeneity, and unrepeatability (20, 21). Therefore, the development of a novel, accurate, and noninvasive method for PD-L1 and TMB assessment is appealing for clinical practice.

Radiomics is inspired by the combination of artificial intelligence and medical imaging. High-throughput and quantitative imaging features reflect the underlying pathophysiology and reveal information on tumor phenotypes (22, 23). Computed tomography (CT) is routinely used for tumor staging and diagnosis in clinical practice. Previous results showed that computed tomography radiomics analysis (CTRA) could be applied in the prediction of gene mutations and tumor phenotypes. Most radiomics research focused on epithelial growth factor receptor (EGFR) (24) and anaplastic lymphoma kinase (ALK) mutations (25), and only a few studies mentioned PD-L1 expression or TMB status in patients treated with chemotherapy (26, 27). Theoretically, the tumor phenotype provided by CT hides a potential correlation with PD-L1 and TMB expression status, which can be quantitatively analyzed. Therefore, the present study compared the performance of the radiomics signature and pretreatment clinical and morphological factors in predicting PD-L1 and TMB status, then developed and validated optimal predictive models to identify patients who may benefit from immunotherapy.

## Materials and Methods

### Patient Selection

For this study, a total of 120 patients were retrospectively enrolled from January 2017 to October 2019 at Shandong Provincial Hospital Affiliated to Shandong First Medical University. The following inclusion criteria were used: 1) pathological diagnosis of lung adenocarcinoma *via* biopsy or bronchofiberoscopy; 2) clinical stages III–IV according to the eighth edition the American Joint Committee on Cancer (AJCC) using pretreatment CT; 3) PD-L1 expression level was tested by immunohistochemistry (IHC); 4) no antitumor therapy received; and 5) sufficient tumor tissue for IHC staining to evaluate PD-L1 expression level and NGS to detect TMB status. Clinicopathological and CT morphological variables were collected in accordance with the protocol and are detailed in **Table 1**.

**Table 1:** The clinicopathological and morphological factors of patients with NSCLC in the training dataset and validation dataset.

Factors	Training	Validation	p
**Age**	63 (49–78)	62 (48–77)	0.711
**Gender**			0.274
Male	54	22	
Female	36	8	
**Smoking**			0.830
Yes	53	19	
No	37	11	
**ECOG PS**			0.143
0–1	72	20	
2	18	10	
**Stage**			0.661
T3	61	19	
T4	29	11	
**Shape**			0.086
Round	57	13	
Irregular	33	17	
**Location**			0.503
Central	58	22	
Peripheral	32	8	
**Speculation**			0.673
Yes	35	13	
No	55	17	
**Cusp angle**			0.313
Yes	18	9	
No	72	21	
**Vacuole sign**			0.286
Yes	34	15	
No	56	15	
**Pleural indentation**			0.391
Yes	31	13	
No	59	17	
**Vascular convergence**			0.527
Yes	52	15	
No	38	15	
**Differentiation Degree**			0.137
Well	24	10	
Median	41	17	
Poor	25	3	

ECOG PS, Eastern Cooperative Oncology Group Performance Status; NSCLC, non-small cell lung cancer.

The Research Ethics Committee of Shandong Provincial Hospital Affiliated to Shandong First Medical University approved this study. All protocols were performed in accordance with the guidelines and ethical principles stated in the 1964 Helsinki declaration. Informed consent was obtained from all participants.

### Immunohistochemistry Staining and Next-Generation Sequencing Assay

Formalin-fixed paraffin-embedded (FFPE) samples from NSCLC patients were sliced at a thickness of 3–4 µm. Unstained sections were de-waxed in xylene, rehydrated in a series of ethanol solutions, and subjected to antigen retrieval in a microwave under middle-to-high pressure for 5 min. Sections were stained with an anti-PD-L1 [VENTANA, clone(c): sp-263] primary antibody in a humidified chamber at 37°C for 60 min then incubated with secondary anti-rabbit and anti-mouse antibodies (Zhongshan Golden Bridge Biotechnology Company) at 37°C for 15 min. Subsequently, 3’3’-diaminobenzidine (DAB) was used to visualize PD-L1 staining. Slides were counterstained with hematoxylin and differentiated with acid alcohol (28). As a previous study stated, we defined “PD-L1 expression positive” as 50% or more viable tumor cells exhibiting membrane staining with any intensity [tumor proportion score (TPS) ≥50%] (29).

We followed the conventional method, and tumor DNA was isolated from FFPE tumor sections. The tumor samples were subjected to NGS using capture panels representing 1,024 cancer-related genes in Gene^+^OncoMDR, which was performed on HiSeq NGS platforms (Illumina Inc., San Diego, CA, USA). TMB was calculated as the total number of mutations counted divided by the size of the coding region of the targeted territory per Mb. Alternations that were known as germline polymorphisms and oncogenic drivers were excluded. According to a related study, the median score was the cutoff value of 4/Mb (30). The variation data reported in this paper have been deposited in the Genome Variation Map (GVM) in Big Data Center, Beijing Institute of Genomics (BIG), Chinese Academy of Science, under accession number GVM000133.

### Region of Interest Segmentation and Feature Extraction

All patients underwent pretreatment contrast-enhanced diagnostic chest, abdomen, and neck CT. CT images were obtained using a 256 detector row CT scanner (Phillips, Netherlands). The image parameters were as follows: tube rotation time 0.5 s; voltage of 110–120 Kvp; tube current of 150–200 mA; and reconstruction slice thickness of 2.5 mm with standard soft-tissue algorithm reconstruction.

CT images were imported into 3D Slicer software edition 4.7 (Harvard, USA) and read with lung (1,500/-500 Hu) and mediastinal (300/-60 Hu) window settings. Tumor segmentation was performed to select primary lesions of NSCLC cases after image acquisition. Two independent oncologists with 10 years and 15 years of experience who were blinded to the clinical data manually contoured the region of interest (ROI). Consensus was reached *via* discussion when interobserver variability was apparent.

A total of 462 quantitative feature extractions were performed by the open-source Imaging Biomarker Explorer software (IBEX, MD Anderson, Houston, TX, USA) and categorized into five subtypes: 1) first-order features; 2) size and shape features; 3) histogram intensity features; 4) texture features involving gray-level co-occurrence matrix (GLCM), gray-level run length matrix (GLRLM), and gray-level size zone matrix (GLSZM); and 5) wavelet features. Briefly, the first-order features described the distribution of voxel intensities. Size and shape features described the morphological structure of the lesion. Histogram intensity features characterized the distribution of voxel intensities in the tumor. Texture features (GLCM, GLRLM, and GLSZM) conveyed information on the spatial relationships between voxels. Wavelet features provided a tractable method of decomposing features into different frequency sub-bands, performing intensity and texture features derived from wavelet transformations of CT images (**Supplementary File 1**).

To improve texture discrimination, all radiomics features were subjected to z-score normalization and transformed to a mean of 0 and a standard deviation of 1. Thirty patient images were randomly selected for reproducibility testing using inter-observer and intra-observer assessments. Tumor segmentation by two oncologists was the inter-observation, and one radiologist’s repeating of the tumor contouring was the intra-observation. Features with inter-/intra-class correlation coefficients (ICCs) ≥0.8 were considered robust and selected for further analysis.

### Feature Selection and Radiomics Signature Building

The present study used a machine-learning method for feature selection in R3.4.2 (Auckland, New Zealand). To minimize overfitting and selection bias, the least absolute shrinkage and selection operator (LASSO) algorithm was performed for the regression of high-dimensional data. The LASSO regression model was conducted by 10-fold cross-validation based on the minimum criteria using the *glmnet* package in R software (31) (version 3.4.2, http://www.r-project.org/) (**Supplementary File 2**). The likelihood ratio test was used for backward stepwise selection, which used Akaike’s information criterion (AIC) as the stopping rule. The radiomics signatures (Rad-score) were calculated as a linear combination of the selected features.

### Statistical Analysis

Combined models on the basis of clinical, CT morphological, and radiomics features were developed by binomial logistic regression for PD-L1 expression and TMB status. The chi-square test or Fisher’s test was employed to analyze categorical variables. The Mann–Whitney U-test was used to compare the continuous variables between groups. The predictive value was assessed by the area under the curve (AUC) of the receiver operating characteristic (ROC) curve analysis using the *pROC* package in R software (32). A two-sided p-value <0.05 was considered a significant difference.

## Results

**Figure 1:**
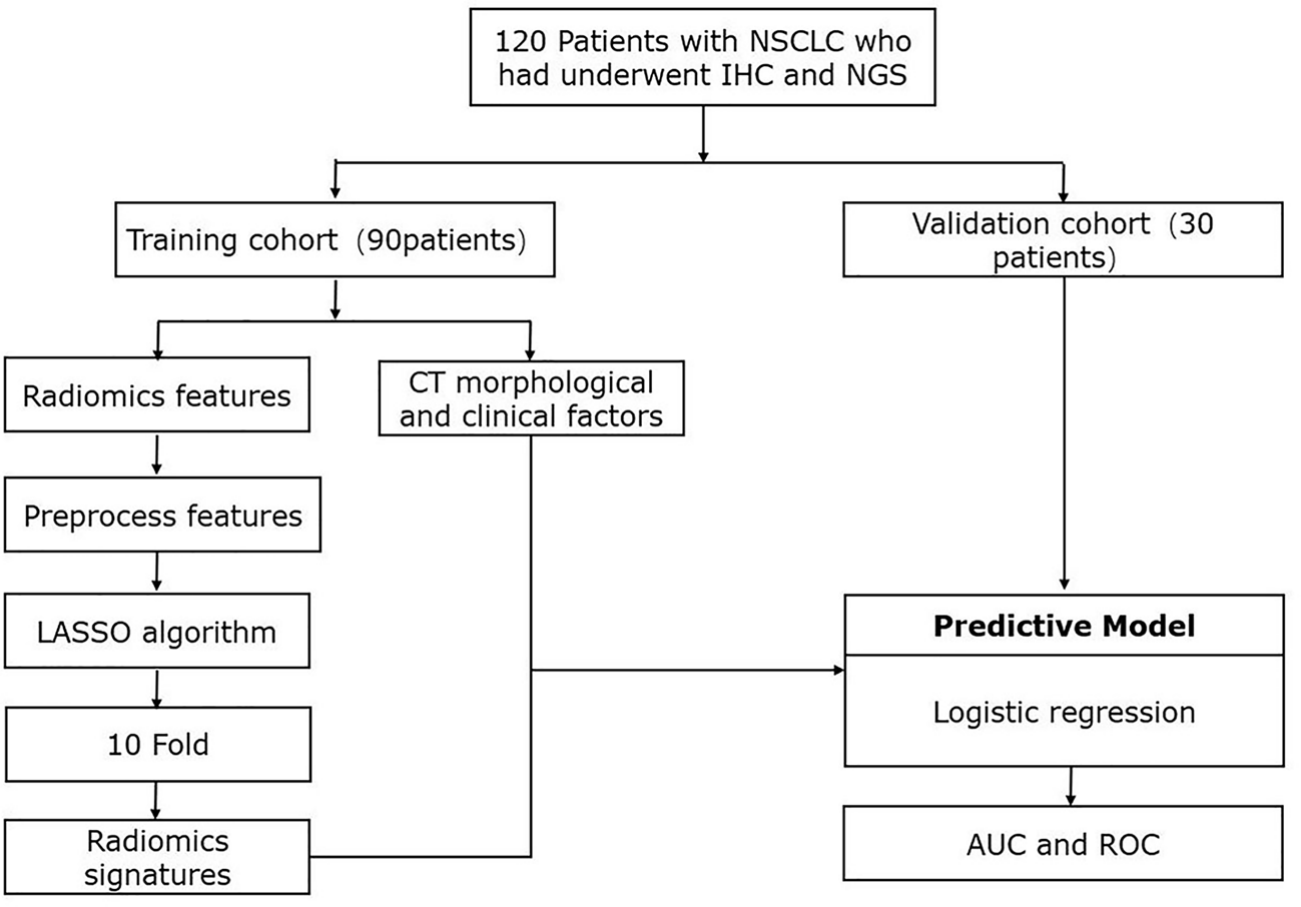
Workflow of study design and radiomics process. IHC, immunohistochemistry, immunobiological staining; NGS, next-generation sequencing; CT, computed tomography; LASSO, least absolute shrinkage and selection operator; AUC, area under the curve; ROC, receiver operating characteristic.

A total of 120 patients with NSCLC were included in this retrospective analysis. The flowchart of the research is presented in **Figure 1**. Patients were randomly divided into a training dataset (90 patients) or a validation dataset (30 patients). The clinicopathological and CT morphological variables are summarized in **Table 1**. There were no significant differences between these factors in the training or validation datasets, including age, differentiation level, staging, and other variables.

The results of clinicopathological and CT morphological factors between patients with positive/negative PD-L1 expression and high/low TMB status in the training set are listed in **Tables 2** and **3**. The differentiation degree (p = 0.005) and tumor shape (p = 0.006) were associated with PD-L1 expression. PD-L1-positive expression was found more frequently in patients with vascular convergence (p = 0.007). We did not determine the predictive values of smoking status (p = 0.437), cusp angle (p = 0.787), or other factors for PD-L1 expression. Staging (p = 0.023) and differentiation degree (p = 0.017) showed statistically significant differences in the identification of TMB status. Higher TMB status tended to correlate with vacuole signs (p = 0.016). There was no discrepancy in TMB status by tumor location (p = 0.509). The clinical models were generated on the basis of multivariate regression analysis, which showed moderate performance for the classification of PD-L1 and TMB with AUCs of 0.650 and 0.661, respectively.

**Table 2:** The correlation of PD-L1 expression level and clinicopathological with CT morphological factors.

Factors	Positive	Negative	p
**Age**	62 (49–78)	63 (50–77)	0.562
**Gender**			0.263
Male	32	22	
Female	26	10	
**Smoking**			0.437
Yes	35	18	
No	23	14	
**ECOG PS**			0.417
0–1	48	24	
2	10	8	
**Stage**			0.059
T3	35	26	
T4	23	6	
**Shape**			0.006
Round	43	14	
Irregular	15	18	
**Location**			0.503
Central	36	22	
Peripheral	17	15	
**Speculation**			0.102
Yes	19	16	
No	39	16	
**Cusp angle**			0.787
Yes	11	7	
No	47	25	
**Vacuole sign**			0.496
Yes	20	14	
No	38	18	
**Pleural indentation**			0.069
Yes	24	7	
No	34	25	
**Vascular convergence**			0.007
Yes	40	12	
No	18	20	
**Differentiation Degree**			0.005
Well	20	4	
Median	28	13	
Poor	10	15	

ECOG PS, Eastern Cooperative Oncology Group Performance Status.

**Table 3:** The correlation of TMB status and clinicopathological with CT morphological factors.

Factors	High	Low	p
**Age**	63 (48–76)	63 (49–78)	0.427
**Gender**			0.519
Male	29	25	
Female	16	20	
**Smoking**			0.086
Yes	31	22	
No	14	23	
**ECOG PS**			0.187
0–1	39	33	
2	6	12	
**Stage**			0.023
T3	25	36	
T4	20	9	
**Shape**			0.382
Round	26	31	
Irregular	19	14	
**Location**			0.509
Central	31	27	
Peripheral	14	18	
**Speculation**			0.666
Yes	19	16	
No	26	29	
**Cusp angle**			0.430
Yes	11	7	
No	34	38	
**Vacuole sign**			0.016
Yes	23	11	
No	22	34	
**Pleural indentation**			0.078
Yes	21	10	
No	28	31	
**Vascular convergence**			0.137
Yes	32	20	
No	13	25	
**Differentiation Degree**			0.017
Well	6	18	
Median	24	17	
Poor	15	10	

ECOG PS, Eastern Cooperative Oncology Group Performance Status; TMB, tumor mutation burden.

The radiomics analysis contained 462 features; all of them were extracted from the segmented pretreatment CT images. After robust and reproducibility tests, 238 out of 462 features were preserved for further analysis with an ICC greater than 0.8. To construct the radiomics signatures for PD-L1 and TMB assessment, six and five features were respectively filtered in the training cohort with non-zero coefficients in the LASSO logistic regression model. LASSO was performed using 10-fold cross-validation based on the minimum criteria. The selected features are listed in **Table 4**. PD-L1 expression prediction using the above radiomics signature showed a favorable assessment efficacy with an AUC of 0.730, and the AUC was 0.759 for discriminating the mutation status of TMB.

**Table 4:** The predictive values of radiomics features for PD-L1 expression levels and TMB status.

Features	PD-L1			
	Class	AUC	95% CI	p
Kurtosis	Histogram	0.585	0.473–0.691	0.033
ClusterTendency	GLCM	0.624	0.550–0.698	0.005
SizeZoneNonUniformity	GLSZM	0.638	0.477–0.799	0.012
GrayLevelNonUniformityNormalized	GLRLM	0.704	0.672–0.737	<0.001
HLH-LongRunHighGrayLevelEmphasis	Wavelet	0.695	0.586–0.790	0.006
HLL-HighGrayLevelZoneEmphasis	Wavelet	0.693	0.582–0.802	<0.001
**Features**	**TMB**			
	**Class**	**AUC**	**95% CI**	**p**
InterquartileRange	Histogram	0.733	0.562–0.864	<0.001
GrayLevelNonUniformity	GLRLM	0.645	0.471–0.794	0.004
MaximumProbability	GLCM	0.588	0.416–0.747	0.032
LHL-AverageIntensity	Wavelet	0.521	0.350–0.688	0.027
HLL-RobustMeanAbsoluteDeviation	Wavelet	0.650	0.571–0.729	<0.001

AUC, area under the curve; GLCM, gray-level co-occurrence matrix; GLRLM, gray-level run length matrix; GLSZM, gray-level size zone matrix; PD-L1, programmed death-ligand 1; TMB, tumor mutation burden.

**Figure 2:**
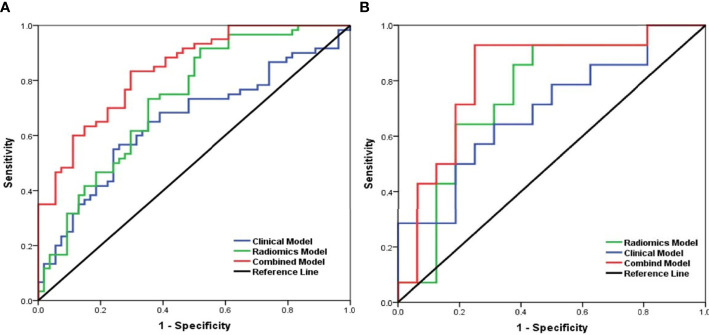
Receiver operating characteristic (ROC) curves of the biomarkers for classifying programmed death-ligand 1 (PD-L1) expression level based on clinical factors alone (blue), radiomics features alone (green), and a combined model that combined clinical and radiomics features (red) in the training set **(A)** and validation set **(B)**.

**Figure 3:**
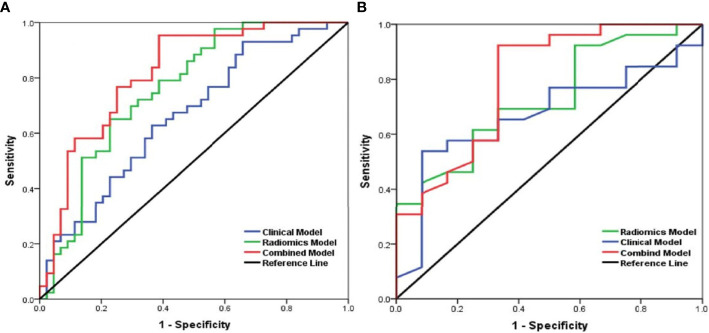
Receiver operating characteristic (ROC) curves of the biomarkers for tumor mutation burden (TMB) status prediction based on the clinical model (blue), radiomics model (green), and a combined model that combined clinical and radiomics features (red) in the training set **(A)** and validation **(B)** set.

According to multivariate analysis, the combined models were constructed using a combination of the clinicopathological, CT morphological factors, and radiomics signatures. ROC analysis of the training dataset demonstrated that we improved the predictive efficacy of PD-L1 expression compared to the radiomics signature and clinical model alone, with an AUC of 0.839, sensitivity of 0.917, and specificity of 0.481. The combined model maintained a high predictive value in the validation dataset with an AUC of 0.793, sensitivity of 0.894, and specificity of 0.502 (**Figure 2** and **Table 5**). Compared to the radiomics signature or clinical model, the integrated model showed better performance for TMB status prediction according to the increased AUC = 0.818, sensitivity = 0.953, and specificity = 0.614. As the results demonstrated, an obvious separation between high and low TMB status was detected in the validation dataset with an AUC = 0.786 (**Figure 3** and **Table 6**).

**Table 5:** The predictive performance of the radiomics model, the clinical model, and the combined model for predicting PD-L1 expression levels in the training and validation sets.

PD-L1	Training			
	AUC	95% CI	Sensitivity	Specificity
**Radiomics**	0.730	0.637–0.823	0.833	0.704
**Clinical**	0.650	0.549–0.751	0.550	0.759
**Combination**	0.839	0.769–0.909	0.917	0.481
	**Validation**			
	**AUC**	**95% CI**	**Sensitivity**	**Specificity**
**Radiomics**	0.722	0.625–0.819	0.794	0.692
**Clinical**	0.645	0.505–0.785	0.583	0.712
**Combination**	0.793	0.712–0.874	0.894	0.502

AUC, area under the curve; PD-L1, programmed death-ligand 1.

**Table 6:** The predictive performance of the radiomics model, the clinical model, and the combined model for predicting TMB status in the training and validation sets.

TMB	Training			
	AUC	95% CI	Sensitivity	Specificity
**Radiomics**	0.759	0.657–0.861	0.830	0.636
**Clinical**	0.661	0.547–0.775	0.651	0.773
**Combination**	0.818	0.728–0.908	0.953	0.614
	**Validation**			
	**AUC**	**95% CI**	**Sensitivity**	**Specificity**
**Radiomics**	0.731	0.632–0.830	0.784	0.649
**Clinical**	0.639	0.513–0.765	0.667	0.674
**Combination**	0.786	0.713–0.859	0.879	0.512

AUC, area under the curve; TMB, tumor mutation burden.

## Discussion

With the evolution of immune therapy, predictive biomarkers have become increasingly important in the guiding of NSCLC treatment according to the latest National Comprehensive Cancer Network (NCCN) guidelines. The present study developed pretreatment CT-based radiomics signatures to effectively classify positive/negative PD-L1 expression levels and high/low TMB status in NSCLC patients. The combination model AUCs were higher than the clinical models and radiomics signatures alone, which indicated that it was a reliable means of identifying patients who may benefit from immunotherapy at diagnosis. The conclusions were consistent with the independent validation cohort. To our knowledge, few related studies referred to pretreatment CT-based radiomics of PD-L1 and TMB in advanced-stage NSCLC patients.

The present research demonstrated that PD-1 and its ligand PD-L1 were confirmed as the most important breakthrough targets in the development of effective ICB immunotherapy. PD-1 is expressed on T cells, B cells, NK cells, and tumor-infiltrating lymphocytes (TILs). Velcheti et al. (33) showed PD-L1 expression on various types of cancer cells. The PD-1/PD-L1 pathway-induced downregulation of T cells, apoptosis, and exclusion from the tumor microenvironment resulted in immune escape (34). Cancer cells upregulate PD-L1 in response to the immune system and form a suitable immune suppression microenvironment for proliferation (35). Investigations on the mechanism of PD-1/PD-L1 pathway induction of immune escape are ongoing, which provide a theoretical foundation and clinical direction for further immunotherapy. More specifically, PD-1/PD-L1 induces a conformational change in PD-1 that contributes to phosphorylation of the cytoplasmic immunoreceptor tyrosine-based inhibitory motif (ITIM) and the immunoreceptor tyrosine-based switch motif (ITSM) by Src family kinases (36). These phosphorylated tyrosine motifs subsequently recruit protein tyrosine phosphatases [src homology 2 (SH2) domain-containing tyrosine phosphatase 2 (SHP-2) and SH2 domain-containing tyrosine phosphatase 1 (SHP-1)] to attenuate T cell-activating signals. Otherwise, PD-L1 also interacts with CD80, which triggers inhibitory signals to activated T cells (37). Blockade of the PD-1/PD-L1 interaction using monoclonal antibodies would bring considerable survival benefit and produce a durable response in NSCLC (38). Various studies in NSCLCs reported 20%–25% and even 80% objective response rates for PD-1/PD-L1 monoclonal antibodies (39). Gong et al. (40) reported that the downregulation of PD-L1 could reduce radiation resistance by promoting apoptosis. The combination of PD-L1 antibodies and radiotherapy synergistically promoted antitumor immunity by increasing CD8 T-cell infiltration and decreasing the accumulation of myeloid-derived suppressor cells (MDSCs) and tumor-infiltrating regulatory T cells (iTregs) in a mouse model (40). PD-L1 may be used as a biomarker in NSCLC patients with high expression (≥50%). The Food and Drug Administration (FDA) approval for pembrolizumab in NSCLC was received in October 2014 (41).

Although ICB has been proven successful, some major challenges still need to be overcome, involving drug resistance, low response rate, and immune-related adverse events. The mechanism of immunotherapy for NSCLC remains indistinct and indefinite. As an indicator for ICB treatment, PD-L1 expression varies between tumor stages, cases, and samples, and information on the molecular regulation of PD-1/PD-L1 is limited. Certain oncogenic signaling pathways may promote tumor growth by driving PD-L1 expression, which leads to immune evasion. Evidence obtained in the past few years has shown that oncogenic signals derived from basic transcription factors, effector elements of signaling pathways, and changes in upstream receptor activity affected the expression of PD-L1.

The first evidence that the oncogenic activation of the mitogen-activated protein kinase (MAPK) pathway was associated with immune evasion of NSCLC cells came from the discovery that treatment with mutant BRAF inhibitors led to increased T-cell infiltration and the downregulation of PD-L1 expression in the melanoma microenvironment (42). The MAPK pathway was also involved in the upregulation of PD-L1 in tumor cells in response to chemotherapy drugs (43). For example, a MEK inhibitor blocked paclitaxel-induced PD-L1 expression, and a low concentration of cisplatin stimulated the expression of PD-L1 *via* MAPK activation. Furthermore, several studies confirmed the role of the phosphoinositide 3-kinases (PI3Ks)/protein kinase B (Akt) pathway in the regulation of PD-L1 in cancer cells. PI3K inhibitors acted on tumor cells that were resistant to BRAF inhibitors and resulted in a decrease in PD-L1 expression. Studies showed that probably the PI3K/Akt pathway regulated PD-L1 expression by either transcriptional or posttranscriptional mechanisms in a cell and tissue type-dependent manner (44). Inhibition of Akt led to decreased PD-L1 expression, and its downstream effector mammalian target of rapamycin (mTOR)/S6 did not mediate Akt-induced PD-L1 expression (45). Despite mTOR, another downstream target of Akt, nuclear factor kappa B (NF-ĸB) regulated PD-L1 expression. It had been shown that Akt by activation of NF-κB upregulates the expression of PD-L1.

Hypoxia-inducible factor (HIF)-1 regulated PD-L1 *via* binding to the PD-L1 promoter hypoxia response element (HRE) site to promote PD-L1 transcription in tumor cells (TCs) and the tumor microenvironment (46). There were two HRE-binding sites, HRE-1 and HRE-4; among them, HRE-4 has higher affinity to HIF-1 than HRE-1. NF-ĸB was a common transcriptional factor and had been shown to be involved in the regulation of PD-L1. Fang et al. (47) pointed out that NF-ĸB regulated Epstein–Barr virus latent membrane protein 1 (LMP1)-induced PD-L1 expression as the caffeic acid phenethyl ester, which decreased the induction of PD-L1 expression. NF-ĸB also had a main role in interferon proteins (IFN)-γ-induced PD-L1 expression. Signal transducer and activator of transcription 3 (STAT3) had also been demonstrated to regulate PD-L1 *via* binding with the PD-L1 promoter and regulating its transcription. Marzec et al. (48) suggested that STAT3 regulated PD-L1 and its transcription *via* binding to the PD-L1 promoter.

The role of miRNAs in the upregulation or downregulation of PD-L1 expression was revealed recently. This regulation may involve direct binding to PD-L1 mRNA or indirectly affect the expression of other PD-L1 regulatory factors. MiR-34a, which binds to the 3’ untranslated region (UTR) of PD-L1, reduced PD-L1 mRNA levels in NSCLC cells (49). P53 may inhibit the expression of PD-L1 *via* miR-34 in NSCLC. Previous research reported that IFN-γ suppressed miR-513 expression, and the overexpression of miR-513 blocked IFN-γ-induced PD-L1 expression (50). In contrast, tumor necrosis factor (TNF)-α and IFN-γ induced miR-155 and inhibited PD-L1 expression (51). Both miR-513 and miR-155 may be considered a system to fine-tune PD-L1 expression upon IFN-γ signaling. MiRNAs also influenced PD-L1 expression in an indirect manner. MiR-197 suppressed PD-L1 expression *via* its direct action on the CKS1B-STAT3 cascade in NSCLC. Exosomes derived from NSCLC cells carrying low levels of miR-34c-3p could be transported into the cytoplasm of NSCLC cells and accelerate NSCLC invasion and migration by upregulation of integrin α2β1. MiR-34c-3p can be a diagnostic and prognostic marker for NSCLC. We trust MiR-34c-3p might be considered as a therapeutic target for NSCLC (52).

There is no doubt about the crucial role of PD-L1 expression status in predicting the checkpoint inhibitor response and prognosis in patients with NSCLC, but few studies have linked radiomics features extracted from pretreatment CT to the prediction of PD-L1 expression. A recent study evaluated PD-L1 expression based on PET/CT images in patients with NSCLC. CT radiomics signatures for PD-L1 expression over 1% or 50% scored 0.86 and 0.91, respectively, which were much higher than radiomics signatures based on PET and PET/CT (26). First, the predictive model did not include clinical factors or morphological features. Second, the sample size was too small, and the construction model of PD-L1 expression from radiomics features was not robust. Third, it was obvious that the PET imaging resolution and definition were lower than those in CT, which contributed to the limited number of features and an uncertain result. Yoon et al. (53) inferred that a combined predictive model that used clinical and radiomics features (AUC = 0.646) showed better performance than the clinical model alone (AUC = 0.550). Their conclusion is consistent with our AUC = 0.784, but the predictive efficacy of their model was much lower.

Positive PD-L1 expression level could be performed as an indicator of response rate specifically for adenocarcinoma patients, and it was even better than chemotherapy (54). However, some reports challenged this conclusion and stated that patients with negative PD-L1 expression also responded to immunotherapy with a 0%–17% objective response rate (55). Otherwise, the evaluated methods of PD-L1 expression were limited, and the cutoff values of positive PD-L1 expression are variable in some literature (56, 57). Therefore, the identification of another treatment decision-related biomarker is a priority for antitumor immunotherapy. A robust response to checkpoint inhibitors was reported in NSCLC, gastric cancer, and melanoma with higher TMB status. High TMB status may result in more neoantigens, which are much more easily recognized by the immune system and more sensitive to anti-PD-L1 antibodies. A total of 240 patients with advanced NSCLC were assessed recently for TMB according to the median count of 7.4/Mb (15), but a median mutation TMB was determined to be 9.9/Mb in patients who received atezolizumab in Kowanetz et al. (58). Due to filtering methods and diverse NGS panel contents, it was challenging to compare TMB results between these studies. The threshold of TMB must be validated and evaluated in further investigations using standard and uniform procedures.

The genomic heterogeneity of malignant tumors contributes to regional variations in stromal structure and may be described as an imaging phenotype by features (59). The information on PD-L1 expression and TMB status is commonly obtained from biopsy or resected tissues and assessed using IHC and NGS. Compared to traditional techniques, radiomics can noninvasively evaluate and objectively reflect tumor heterogeneity. Jiang et al. (26) reported that CT- and PET-based radiomics signatures showed good performance for distinguishing various expression degrees of PD-L1. TMB and somatic driver mutations, such as EGFR/P53, were identified using CT radiomics parameters based on a machine-learning technique, as O’Connor et al. (60) proposed. To date, only two prior studies investigated the role of CT radiomics in decoding TMB status. A previous study indicated that quantified radiomics features of lesions were associated with TMB after analysis of TMB data from 327 patients with NSCLC (61). Wang et al. (30) evaluated 61 endometrial tumors in 51 patients and demonstrated an AUC of 0.671 for assessing TMB using a clinical–radiomics model. Compared to the results of Wang et al. (30), our results revealed that the multimodality model predicted TMB status more accurately. Therefore, the predictive model constructed in the present study may provide sufficient information for future therapy.

The hypothesis supporting the use of pathological phenotype and radiomics in medicine is related to the underlying biological processes. The procedures of medical imaging and the development of high-throughput algorithms to extract quantitative features from images have contributed to the improvement of radiomics, which provide more information on biological characteristics compared to the visual interpretation of the image as a picture (62). Radiomics and pathology fill the need to assess tumor heterogeneity. The detection of tumor phenotype heterogeneity is one of the main goals of novel therapeutic strategies, and the utility of blood markers might present disadvantages that are overcome with the use of radiomics and pathology (63).

We verified the stability of the model, and the results showed that radiomics and combination models had good reliability. In the training and validation datasets, the predictive accuracy, specificity, and sensitivity resulting from 10-fold cross-validation were greater than 0.7, which means that the results of the model were not caused by overfitting. Kurtosis is a measure of the “peakedness” of the distribution of ROIs. A lower value indicates that the mass of the distribution is primarily toward a spike near the mean value. Cluster tendency describes groupings of voxels with similar gray-level values. SizeZoneNonUniformity and GrayLevelNonUniformity variables were used to assess the variability of size zone volume and gray-level intensity values in the image, and lower values for both variables suggest lower heterogeneity of size zone volumes and intensity values (64). In contrast, gray-level nonuniformity normalization reflects the similarity of the gray-level intensity values, and a lower value is associated with a greater similarity in intensity values. Moreover, HLH-LongRunHighGrayLevelEmphasis and HLL-HighGrayLevelZoneEmphasis were the means of the distribution of the long homogeneous runs with high gray levels and the distribution of the high gray-level zones with wavelet transformation. InterquartileRange represents the range between the 25th and 75th percentiles of the CT image array. Maximum probability was defined as the most predominant pair of neighboring intensity values. HLL-RobustMeanAbsoluteDeviation was used to evaluate the distance of all intensity values from the mean value. Our results clearly demonstrated that histopathological heterogeneity correlated with the radiomics features of CT images. Quantification of the spatial complexity of tumor medical images reveals the spatial complexity in pathology and the phenotypic intertumoral heterogeneity. Consistent with Choi et al. (65), radiomics features may actually reflect spatial heterogeneity in the ROI of the tumor.

In the traditional morphological features of NSCLC lesions, we also reported that PD-L1 was statistically associated with vascular convergence, and this conclusion was consistent with a previous study (66). In addition to vascular convergence, some researchers stated that cavitation or pleural indentation was a surrogate indicator of PD‐L1 positivity and correlated with the pathological invasiveness of the malignant nodule (67). However, none of the remaining morphological features was related to PD-L1 expression. A reasonable explanation for this result is the clinical characteristics of the study population. Most previously studied cases were early stage and resectable (68), and these tumor morphological and biological features are vastly different from our tumors. In summary, tumor phenotype was related to radiomics features and was reflected in the radiological features. However, more factors must be discussed.

Some clinical factors had predictive power, and this finding suggests that these variables are relevant to genotype but are not determining factors. Our multivariate analysis demonstrated that differentiated grade was related to TMB. There was no evidence of an association between TMB and somatic mutation burden, but it is likely that tumors with a high somatic mutation burden are poorly differentiated. This conclusion was reached from recent research of a higher EGFR mutation in well-differentiated tumors, which may be due to a reduction in TMB associated with a single driver gene mutation (69). Another finding of our study was PD-L1 expression heterogeneity. High PD-L1 expression was detected in poorly histological patterns, and similar results were mentioned previously (70, 71). Because of PD-L1 expression heterogeneity, we should select a representative slice containing the most diverse histological subtypes. These factors were characteristics of instability and uncertainty, and it was necessary to add radiomics signatures to a clinical model to improve the predictive efficacy in NSCLC.

There were some limitations in this study. First, it was a retrospective study, and selection bias was inevitable. Prospective studies are required to investigate and validate this hypothesis in a larger sample size. Second, CT images were acquired from one machine at a single center, which resulted in conclusions that are hardly generalizable to other study centers. Multicenter and outside databases are essential in future research. The third shortcoming was that oncologists manually contoured all ROIs. However, we evaluated the ICC *via* inter-/intra-observations, which effectively reduced the likelihood of deviations. Last, it would be better to examine whether radiomics features were associated with survival following therapy with checkpoint inhibitors in NSCLC patients.

## Conclusion

In summary, the quantitative radiomics features extracted from pretreatment CT were noninvasively associated with specific PD-L1 expression and TMB status. The combination of clinical factors and radiomics signatures significantly improved the predictive performance. Our findings suggest a promising future for the guiding of immunotherapy in NSCLC patients and deserve further in-depth study.

## Data Availability Statement

The variation data reported in this paper have been deposited in the Genome Variation Map (GVM) in Big Data Center, Beijing Institute of Genomics (BIG), Chinese Academy of Science, under accession numbers GVM000133 that are publicly accessible at http://bigd.big.ac.cn/gvm/getProjectDetail?project=GVM000133 .

## Ethics Statement

The studies involving human participants were reviewed and approved by Ethics Committee (IRB) at Shandong Provincial Hospital Affiliated to Shandong First Medical University. The patients/participants provided their written informed consent to participate in this study. Written informed consent was obtained from the individual(s) for the publication of any potentially identifiable images or data included in this article.

## Author Contributions

QW designed the study and wrote the manuscript. ZY and QW participated in the study designing and data collection. QL and HD provided the analysis of data and ROI segmentation. AF participated in data collection and offered guidance. ZY carried out the study design and interpretation of data and drafted the manuscript. All authors contributed to the article and approved the submitted version.

## Funding

This study was supported by Shandong Provincial Natural Science Foundation (Grant No. ZR2020QH200) and the special tumor foundation for Scientific Research of Xinda-CSCO (Grant No. 320.6750.19088-88). The funding sources had no role in the study design, data collection, analysis of interpretation, or the writing of this manuscript.

## Conflict of Interest

The authors declare that the research was conducted in the absence of any commercial or financial relationships that could be construed as a potential conflict of interest.

The reviewers JY and QQ declared a shared affiliation with the authors to the handling editor at the time of the review.

## Publisher’s Note

All claims expressed in this article are solely those of the authors and do not necessarily represent those of their affiliated organizations, or those of the publisher, the editors and the reviewers. Any product that may be evaluated in this article, or claim that may be made by its manufacturer, is not guaranteed or endorsed by the publisher.
