# Supplementary material for: Radiomics Study for Predicting the Expression of PD-L1 and Tumor Mutation Burden in Non-Small Cell Lung Cancer Based on CT Images and Clinicopathological Features
Source: Front Oncol. 2021 Aug 6;11:620246. doi: 10.3389/fonc.2021.620246 (PMC8377473; doi:10.3389/fonc.2021.620246)
Supplement: Supplementary file 1 [file DataSheet_1.docx]

| **Size and Shape(16)** | | Maximum3DDiameter, Compactness2, Maximum2DDiameterSlice, Sphericity，MinorAxis, Compactness1, Elongation, SurfaceVolumeRatio, Volume, SphericalDisproportion, MajorAxis, LeastAxis, Flatness, SurfaceArea，Maximum2DDiameterColumn, Maximum2DDiameterRow |
| --- | --- | --- |
| **Histogram Intensity(19)** | | InterquartileRange, Skewness, Uniformity, MeanAbsoluteDeviation, Energy, RobustMeanAbsoluteDeviation, Median, TotalEnergy, Maximum, RootMeanSquared, 90Percentile, Minimum, Entropy, StandardDeviation,, Range，Variance，10Percentile，Kurtosis，Mean |
| **Texture Features** | **GLCM(27)** | SumVariance, Homogeneity1, Homogeneity2, ClusterShade, MaximumProbability, Idmn, Contrast, DifferenceEntropy, InverseVariance, Dissimilarity, SumAverage, DifferenceVariance, Idn，Idm，Correlation, Autocorrelation, SumEntropy, AverageIntensity, Energy, SumSquares, ClusterProminence, Entropy, Imc2, Imc1, DifferenceAverage, Id, ClusterTendency |
|  | **GLRM(16)** | ShortRunLowGrayLevelEmphasis, GrayLevelVariance, LowGrayLevelRunEmphasis, GrayLevelNonUniformityNormalized, RunVariance，GrayLevelNonUniformity, LongRunEmphasis, ShortRunHighGrayLevelEmphasis, RunLengthNonUniformity，ShortRunEmphasis，LongRunHighGrayLevelEmphasis，RunPercentage, LongRunLowGrayLevelEmphasis, RunEntropy，HighGrayLevelRunEmphasis，RunLengthNonUniformityNormalized |
|  | **GLSZM(16)** | GrayLevelVariance，SmallAreaHighGrayLevelEmphasis, GrayLevelNonUniformityNormalized, SizeZoneNonUniformityNormalized, SizeZoneNonUniformity, GrayLevelNonUniformity, LargeAreaEmphasis, ZoneVariance, ZonePercentage, LargeAreaLowGrayLevelEmphasis, LargeAreaHighGrayLevelEmphasis, HighGrayLevelZoneEmphasis, SmallAreaEmphasis, LowGrayLevelZoneEmphasis, ZoneEntropy, SmallAreaLowGrayLevelEmphasis |
| **Wavelet(368)** | | (Histogram Intensity features+ GLCM features) * -LLL，-LLH,-LHL, LHH, -HLL, -HLH, -HHL, -HHH |

**Supplement Files 1. The list of CT radiomics features.**

**Supplement Files 2. The code of LASSO model in R Language.**

# Set your working directory, where your .csv files stored

setwd("C:/Users/wq/Desktop/data/")

# Training data input

train <- read.csv("train.csv")

# Library lasso package

library(glmnet)

# Prepare your training data

x = as.matrix(train[1:90,1:462])

y = train[1:90,463]

# for reproducibility

set.seed(1000)

# LASSO training or fitting

cvfit = cv.glmnet(x, y, nfolds = 10, alpha = 1, family = "binomial",

type.measure = "auc", grouped = TRUE)

# The optimal value of λ

cvfit$lambda.1se

log(cvfit$lambda.1se)

# The actual coefficients at one or more lambda within the range of the sequence,

# and save a .csv file to build signature

coef = coef(cvfit, s = "lambda.1se")

write.csv(as.matrix(coef),"coef.csv")
